# Oncological Outcomes After Okabayashi-Kobayashi Radical Hysterectomy for Early and Locally Advanced Cervical Cancer

**DOI:** 10.1001/jamanetworkopen.2020.4307

**Published:** 2020-05-07

**Authors:** Noriaki Sakuragi, Tatsuya Kato, Chisa Shimada, Masanori Kaneuchi, Yukiharu Todo, Takashi Mitamura, Mahito Takeda, Masataka Kudo, Gen Murakami, Hidemichi Watari

**Affiliations:** 1 Department of Obstetrics and Gynecology, Graduate School of Medicine, Hokkaido University, Sapporo, Japan; 2 Department of Gynecology, Otaru General Hospital, Otaru, Japan; 3 Department of Gynecology, Hokkaido Cancer Center, Sapporo, Japan; 4 Department of Anatomy II, Sapporo Medical University, Sapporo, Japan; 5 Division of Internal Medicine, Jikou-kai Clinic of Home Visits, Sapporo, Japan

## Abstract

**Importance:**

The role of surgery in early-stage cervical cancer has been established, but it is controversial in locally advanced cervical cancer.

**Objective:**

To determine whether a radical hysterectomy method with extended removal of paracervical tissue for locally advanced cervical cancer is associated with satisfactory oncological outcomes.

**Design, Setting, and Participants:**

This retrospective cohort study was conducted from January 1, 2002, to December 31, 2011, and participants were patients with cervical cancer at a single tertiary center in Northern Japan. The median follow-up period was 106 months, and none of the patients were lost to follow-up at less than 60 months. Data analyses were performed from July 1, 2017, to December 31, 2018.

**Exposures:**

Patients underwent radical hysterectomy using the Okabayashi-Kobayashi method. Bilateral nerve preservation was used for stage IB1/IB2 disease and unilateral nerve preservation for stage IIA/IIB if disease extension outside the uterine cervix was 1-sided. Chemotherapy was used as the choice of adjuvant treatment for patients with an intermediate or high risk of recurrence, while some patients chose or were assigned to radiotherapy.

**Main Outcomes and Measures:**

Primary outcomes were the 5-year local control rate and 5-year overall survival rate along with risk factor analysis.

**Results:**

Of 121 consecutive patients, 76 (62.8%) had early-stage cervical cancer in 2008 International Federation of Gynecology and Obstetrics stages IB1 and IIA1 and 45 (37.2%) had locally advanced cervical cancer in stages IB2, IIA2, and IIB. The median (range) age was 42 (26-68) years. Adjuvant radiotherapy was used in 2 patients (3%) with early-stage cervical cancer and 3 (7%) of those with locally advanced cervical cancer. The 5-year local control rates for early-stage cervical cancer and locally advanced cervical cancer were 99% and 87%, respectively. The 5-year overall survival rates for early-stage cervical cancer and locally advanced cervical cancer were 95% and 82%, respectively. Cox regression analysis showed that lymph node metastasis and histology of adeno(squamous)carcinoma were independent risk factors for the overall survival of patients with cervical cancer treated with radical hysterectomy.

**Conclusions and Relevance:**

The nerve-sparing Okabayashi-Kobayashi radical hysterectomy for locally advanced cervical cancer may provide survival not inferior to radical hysterectomy or radiotherapy in published literature. The applicability of radical hysterectomy with adjuvant chemotherapy for locally advanced cervical cancer needs to be validated by prospective comparative trials.

## Key Points

**Question:**

Is radical hysterectomy with extended removal of paracervical tissue associated with satisfactory outcomes for locally advanced cervical cancer?

**Findings:**

This retrospective cohort study of 121 patients with cervical cancer treated with Okabayashi-Kobayashi radical hysterectomy showed that the 5-year local control rates for early-stage IB1/IIA1 disease and locally advanced stage IB2/IIA2/IIB disease were 99% and 87%, respectively, and the 5-year overall survival rates for these groups were 95% and 82%, respectively.

**Meaning:**

Okabayashi-Kobayashi radical hysterectomy with adjuvant chemotherapy may be a treatment option for locally advanced cervical cancer besides radiotherapy/chemoradiotherapy.

## Teaser

This cohort study assesses whether a radical hysterectomy method with extended removal of paracervical tissue for locally advanced cervical cancer is associated with satisfactory oncological outcomes.

## Introduction

Cervical cancer is the fourth most common type of malignant neoplasm in women.^1^ The peak incidence of cervical cancer occurs in the age group of the 30s to 40s in many countries.^2,3,4,5^ Surgery has advantages over radiotherapy because the long-term intractable complications of radiation therapy can be avoided, and ovarian and sexual function in younger patients can be maintained.^6,7^ Radical hysterectomy has been evolving over 100 years since the first description of extended abdominal hysterectomy by Wertheim in 1912.^8^ The Wertheim operation was modified later worldwide.^9,10,11^ The Meigs operation is commonly used in Western countries, and the Okabayashi operation is used in Japan and some areas of Asian countries. The Kobayashi method preserves the pelvic splanchnic nerves and the inferior hypogastric plexus (modified Okabayashi radical hysterectomy).^12,13^ The systematic nerve-sparing procedure opens the tissue plane between the parametrium/paracolpium and the pelvic nerve structures.^14,15,16,17^

Nerve-sparing radical hysterectomy is currently a standard treatment for early-stage cervical cancer (ESCC), which provides for nonbulky (≤4 cm in diameter) stage IB1 and IIA1. Locally advanced cervical cancer (LACC) traditionally included stage IIB to stage IVA. Many oncologists now include stages IB2 and IIA2 disease in this category.^18^ LACC may be divided into stages IB2 to IIB and stages III to IVA. A clear statement about the stages that LACC indicates is necessary. Stage IB2, IIA2, and IIB disease, which are relatively earlier stages in LACC, may be treated with multidisciplinary therapy, including surgery, chemotherapy, and radiotherapy, and these stages may be defined as early locally advanced disease.^19^ The term *LACC*, which is discussed in this article, denotes stage IB2/IIA2/IIB cervical cancer. Little is known about the oncologic validity of nerve-sparing radical hysterectomy in LACC.^20,21^ There are 3 options for treatment of LACC, including concurrent chemoradiotherapy, neoadjuvant chemotherapy followed by surgery, and upfront surgery with or without adjuvant therapy. Concurrent chemoradiotherapy is the treatment of choice for LACC in the National Comprehensive Cancer Network Clinical Practice Guidelines.^22^ In Japan, not only ESCC, but also LACC are treated with radical hysterectomy.^23^ Additionally, radical hysterectomy was used in 46.6% of stage II (substage included 15% of IIA1 and 85% of IIA2 and IIB) cervical cancer in 2015.^24^ The rate of upfront surgery with or without adjuvant therapy for LACC increased from 22.6% to 31.2% in the United States from 2004 to 2012.^19^ The accordance of preoperative diagnosis of parametrial invasion with pathologically confirmed parametrial invasion is as low as 21% to 55%,^25^ and some authors have discussed the role of surgery for LACC from the difficulty in the preoperative diagnosis of stage IIB.^26^ Nerve-sparing radical hysterectomy can be used for the side that is free of tumor invasion in stage II cervical cancer to the extent that it does not decrease the curability of radical hysterectomy.^23^ The current study aimed to determine whether Okabayashi-Kobayashi radical hysterectomy is a useful option for LACC by investigating the patterns of recurrence and long-term survival.

## Methods

### Patients

We included consecutive patients with 2008 International Federation of Gynecology and Obstetrics (FIGO) stages^27^ IB1 to IIB cervical cancer who underwent Okabayashi-Kobayashi radical hysterectomy from January 1, 2002, to December 31, 2011, at Hokkaido University Hospital in Sapporo, Japan. This hospital is a tertiary care center in the central Hokkaido area in Northern Japan, and most of the patients were referred to the hospital from primary care obstetricians and gynecologists and the regional cervical cancer screening center (Hokkaido Cancer Society). There was no selection of patients who may have been more likely to respond to surgery and adjuvant chemotherapy. Anonymized clinical information with the type of treatment and the survival outcomes was registered to the gynecologic cancer registry in Japan Society of Obstetrics and Gynecology.

Patients were followed up every 3 months for the first 3 years, every 6 months in years 4 to 5, and at 12-month intervals after this time. We used chest x-rays and either computed tomography, magnetic resonance imaging, or positron emission tomography scan once a year and at any suspicion of recurrence by bimanual examination, symptoms, and an increase in serum tumor marker that was elevated preoperatively.

Data cleaning and analyses were performed from July 1, 2017, to December 31, 2018. This study was approved by the local ethics committee (institutional review board) of Hokkaido University Hospital, and written informed consent was waived because of the retrospective design. We used the opt-out method on the hospital's website for obtaining consent. The Strengthening the Reporting of Observational Studies in Epidemiology (STROBE) reporting guideline was followed.^28^

### Patient Characteristics

Pathological risk factors were collected and include age, 2008 FIGO stage, histology (squamous cell carcinoma, adeno[squamous]carcinoma), pT classification (tumor pathologically confined to the cervix: pT1b1 and pT1b2; tumor extended outside the cervix: pT2a1, pT2a2, and pT2b), lymph node metastasis (no, yes), lymphovascular space invasion (no, yes), recurrence risk (low, intermediate, high), postoperative adjuvant therapy (no; yes: chemotherapy, radiotherapy), and recurrence (no; yes: local, regional, and distant). The American Joint Committee on Cancer/International Union Against Cancer TNM staging system^29^ was used to describe the extent of cervical cancer. TNM incorporates T (size and extent of the tumor), N (involvement of the regional lymph node), and M (metastasis), which may be determined preoperatively (cTNM or TNM) and postsurgical histologically (pTNM). pT stands for pathologically defined local tumor status. FIGO stages were grouped into the early stage (stage IB1 and IIA1) and locally advanced stage (stage IB2, IIA2, and IIB) for recurrence and survival analyses.

### Nerve-Sparing Radical Hysterectomy

We used systematic nerve-sparing Okabayashi-Kobayashi radical hysterectomy as reported elsewhere.^15,16^ We used bilateral nerve preservation for stage IB1/IB2 disease and unilateral nerve preservation for stage IIA/IIB if disease extension outside the uterine cervix was 1-sided. We did not offer radical hysterectomy to patients with stage IIA/IIB disease with bilateral extension of the tumor.

### Postoperative Adjuvant Therapy

We used postoperative adjuvant treatment according to the recurrence risk after surgery.^23,30^ Intermediate risk was defined as a large tumor size (>4 cm), deep cervical invasion (>2/3), and lymphovascular space invasion. High risk was defined as lymph node metastasis, pathological parametrial invasion, and a positive/close surgical margin. The choice of adjuvant therapy was chemotherapy consisting of paclitaxel and cisplatinum every 3 weeks for 4 to 6 courses.

### Statistical Analysis

The primary outcome measure was the 5-year local control rate and 5-year overall survival. Overall survival included death from any cause. Disease-specific survival included death from cervical cancer. Patients known to be alive or lost to follow-up (the patient was unreachable, and her survival outcome was unknown) at the time of analysis were censored at their last follow-up. The follow-up period was defined as the time from surgery until recurrence or death and the time from surgery until the last confirmation by patient’s visit, telephone, through the nearby affiliated hospital, or direct visit to the patient’s residence.

Recurrence was defined as relapse of tumor after completion of the primary treatment. The site of the recurrence was categorized into local (relapse in the vaginal stump or paravaginal area), regional (relapse in pelvic lymph nodes), and distant (relapse in the area outside the pelvis including paraaortic lymph nodes, distant organs, and peritoneal cavity). Disease-free survival (DFS) was defined as the time from surgery to recurrence.

Five-year local control rate was defined as the rate of patients without local recurrence at 5 years after surgery. We used Fisher exact test to examine the association between the site-specific recurrence and clinical/pathological variables. Competing risks are events that preclude the occurrence of the event in concern or alter the probability of its occurrence.^31^ Regional recurrence, distant recurrence, and death from other causes were considered to be competing risks for local recurrence. We used Gray method^32^ to obtain the first event-specific cumulative incidence curves for local recurrences according to FIGO stage (ESCC vs LACC). We used the Fine-Gray proportional subdistribution hazards regression^33^ to evaluate the association of clinical/pathological variables with the cumulative incidence of local recurrence.

Survival curves were calculated by the Kaplan-Meier method and compared by the log-rank test. We used Cox regression analysis to evaluate the association of clinical/pathological variables with survival. It is generally assumed that 10 outcome events per independent variable are necessary in Cox regression analysis. We performed a univariable Cox regression analysis for overall survival and then obtained a 2-variable regression model based on the number of events we observed.

These statistical analyses were performed using EZR (Saitama Medical Center, Jichi Medical University), which is a graphical user interface for R software, version 3.6.1 (R Foundation for Statistical Computing).^34^ *P *values were 2-sided, and the significance level was set at *P* < .05.

## Results

The clinical characteristics of 121 patients are provided in Table 1, and pathological risk factors are provided in Table 2. The median (range) age for patients was 42 (26-68) years; 50 patients (42%) were younger than 40 years, 36 (30%) were aged 40 to 49 years, and 35 (29%) were older than 49 years. Overall, 76 patients (63%) were at early stage, and 45 (37%) were at locally advanced stage. The recurrence risk was low (45 [37%]), intermediate (43 [36%]), and high (33 [27%]). Among 76 patients with intermediate or high risk for recurrence, 68 patients (89.4%) received adjuvant therapy, of whom 63 received chemotherapy, and 5 underwent radiotherapy. The use of adjuvant chemotherapy in ESCC and LACC was 34% (n = 26) and 82% (n = 37), respectively. The use of adjuvant radiotherapy in ESCC and LACC was 3% (n = 2) and 7% (n = 3), respectively. Among 33 patients with a high risk of recurrence, 8 patients were at extremely high risk (≥5 positive nodes with pathological parametrial invasion). Three of these patients received radiotherapy, and the remaining 5 received chemotherapy.

**Table 1.** Clinical and Demographic Features of Patients With Cervical Cancer Treated With Nerve-Sparing Kobayashi (Modified Okabayashi) Radical Hysterectomy

Characteristic	Patients, No. (%)
Total, No.	121
Age, y	
Median (range)	43.0 (28-68)
<40	50 (42)
40-49	36 (30)
>49	35 (29)
2008 FIGO stage	
Early stage	76 (63)
IB1	72 (60)
IIA1	4 (3)
Locally advanced stage	45 (37)
IB2	13 (11)
IIA2	5 (4)
IIB	27 (22)
pT classification	
pT1	91 (75)
pT1b1	74 (61)
pT1b2	17 (14)
pT2	30 (25)
pT2a1	10 (8)
pT2a2	7 (6)
pT2b	13 (11)
Recurrent risk	
Low	45 (37)
Intermediate	43 (36)
High	33 (27)
Postoperative adjuvant therapy	
None	53 (44)
Chemotherapy	63 (52)
Radiotherapy	5 (4)
Recurrence	
No	103 (85)
Yes	18 (15)
Site of first recurrence	
Local only	6
Regional only	1
Distant only	5
Local and regional	0
Local and distant	1
Regional and distant	5

Abbreviation: FIGO, International Federation of Gynecology and Obstetrics.

**Table 2.** Clinical and Pathological Factors and the Site-Specific Recurrence After Nerve-Sparing Radical Hysterectomy (N = 121)^a^

Clinical and pathological factor	No. (%)	Site-specific recurrence risk					
		Local (n = 7)		Regional (n = 6)		Distant (n = 11)	
		No. (%)	*P* value	No. (%)	*P* value	No. (%)	*P* value
2008 FIGO stage							
Early	76 (63)	1 (1)	.01	3 (4)	.67	4 (5)	.10
Locally advanced	45 (37)	6 (13)		3 (7)		7 (16)	
Histology							
Squamous cell carcinoma	79 (65)	3 (4)	.21	3 (4)	.42	5 (6)	.19
Adeno(squamous)carcinoma	42 (35)	4 (10)		3 (7)		6 (14)	
pT classification							
pT1b	91 (75)	0	<.001	2 (2)	.03	6 (7)	.14
pT2	30 (25)	7 (23)		4 (13)		5 (17)	
Lymph node metastasis							
Negative	88 (73)	0	<.001	3 (3)	.34	5 (6)	.07
Positive	33 (27)	7 (21)		3 (9)		6 (18)	
Lymphovascular space invasion							
Negative	67 (55)	0	.003	4 (6)	.69	6 (9)	>.99
Positive	54 (45)	7 (13)		2 (4)		5 (9)	

Abbreviation: FIGO, International Federation of Gynecology and Obstetrics.

^a^ Fisher exact test was used to examine the association between the site-specific recurrence and clinical/pathological variables.

The median (interquartile range) follow-up period was 106 (70.5-125) months (range, 6-203 months). All survival information was available for all 121 patients for more than 60 months after surgery. No patients were lost to follow-up or censored before 60 months. Fifteen patients (12.4%) died of cervical cancer during 60 months of follow-up, 1 patient (0.8%) died of cervical cancer at 104 months after surgery. One patient (0.8%) died of intercurrent disease at 105 months after surgery, and no patient died of any other causes.

We observed 18 cases of recurrence (15%). Fifteen of 18 cases (83%) occurred within 3 years from surgery, and the remaining 3 (17%) occurred more than 3 years after surgery. The median (range) recurrence-free interval of time from surgery until the diagnosis of local, regional, and distant recurrence was 13 (3-32), 12.5 (3-52), and 20 (3-52) months, respectively. The site-specific recurrence risk for local (n = 7), regional (n = 6), and distant (n = 11) is provided in Table 2, which suggested a different pattern of association between clinical/pathological risk factors and the site-specific recurrence. FIGO stage, pT classification, lymph node metastasis, and lymphovascular space invasion were associated with local recurrence, but histology was not. The association between local recurrence and the factors of pT classification, lymph node metastasis, and lymphovascular space invasion showed complete separation, which may suggest a limitation of the analysis owing to small numbers studied. The association of clinical/pathological risk factors and local recurrence as the first recurrence is provided in Table 3. We observed 1 local recurrence in 72 stage IB1 tumors. None of the stage IB2 and IIA1 tumors were associated with local recurrence, while IIA2 (2 of 5 [40%]) and IIB (4 of 27 [14.8%]) tumors were associated with a high local recurrence rate. Four patients (2 of pT2a1N1M0 and 2 of pT2bN1M0) showed local recurrence in the paracolpium on the same side as the nerve-sparing procedure. The 5-year local control rate for ESCC and LACC was 99% (75 of 76) and 87% (39 of 45), respectively (*P* = .01). The hazard ratio for cumulative local recurrence rate between early and locally advanced disease was 11.2 (95% CI, 1.3-96.0; *P* = .03; Figure, A).

**Table 3.** Evaluation of the Association of Clinical and Pathological Factors With Cumulative Incidence of Local Recurrence After Nerve-Sparing Radical Hysterectomy^a^

Clinical and pathological factor	No. (%)	Local recurrence risk		
		Incidence	Hazard ratio (95% CI)	*P* value
2008 FIGO stage				
Early	76 (63)	1	1 [Reference]	NA
Locally advanced	45 (37)	6	11.5 (1.3-96.0)	.03
Histology				
Squamous cell carcinoma	79 (65)	3	1 [Reference]	NA
Adeno(squamous)carcinoma	42 (35)	4	2.7 (0.6-11.8)	.19
pT classification				
pT1b	91 (75)	0	1 [Reference]	NA
pT2	30 (25)	7	999 500 (467 500-2 137 000)	<.001
Lymph node metastasis				
Negative	88 (73)	0	1 [Reference]	NA
Positive	33 (27)	7	539 000 (251 900-1 153 000)	<.001
Lymphovascular space invasion				
Negative	67 (55)	0	1 [Reference]	NA
Positive	54 (45)	7	100 200 (46 260-217 200)	<.001

Abbreviations: FIGO, International Federation of Gynecology and Obstetrics; NA, not applicable.

^a^ The Fine-Gray proportional subdistribution hazards regression was used for the analysis.

**Figure.**
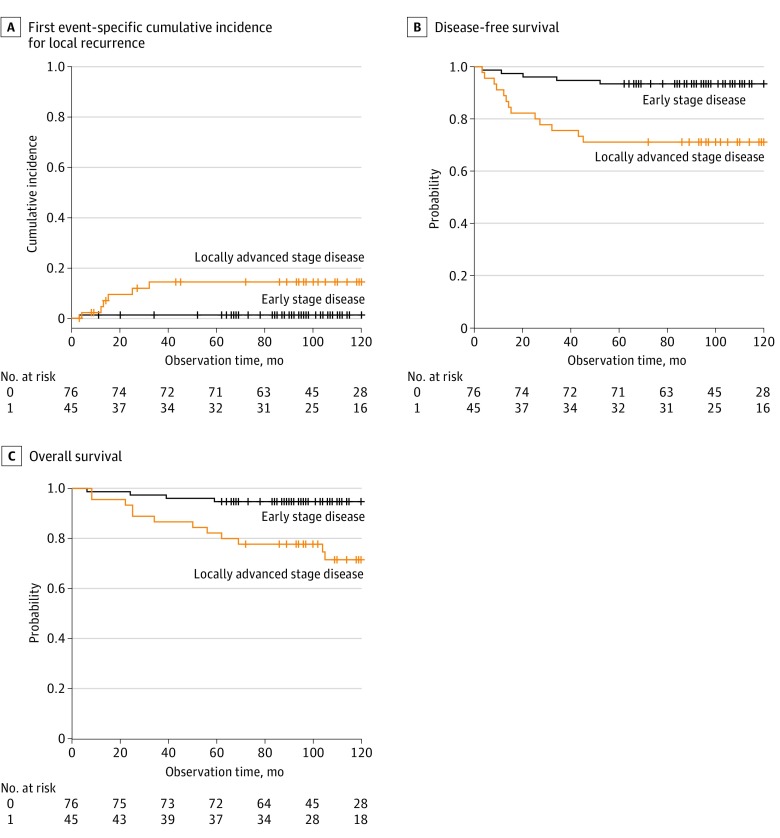
Oncological Outcomes in Early-Stage and Locally Advanced Cervical Cancer Treated With Nerve-Sparing Radical Hysterectomy The cumulative incidence curve for local recurrence (A) was obtained using Gray method. Comparison of the cumulative incidence curves was achieved by the Fine-Gray proportional subdistribution hazards regression. Recurrence in the regional lymph node area and distant organs, and all-cause death were competing risks for local recurrence. Cumulative disease-free survival (B) and cumulative overall survival (C) were obtained using the Kaplan-Meier method, and the association with the Federation of Gynecology and Obstetrics stage was evaluated using the log-rank test. Hazard ratio for disease-free survival and overall survival were calculated using Cox regression analysis.

The cumulative DFS is provided in the Figure, B. The 5-year DFS for all stages combined was 85% (n = 121). The 5-year DFS rate as obtained by the number of patients without recurrence divided by the number of total patients at 60 months after surgery for ESCC and LACC was 71 of 76 (93%) and 32 of 45 (71%), respectively (hazard ratio, 5.0; 95% CI, 1.8-14.1; *P* < .001).

We observed 16 deaths (13%). The cumulative overall survival is provided in the Figure, C. The 5-year overall survival for all stages combined was 90% (n = 121). The 5-year overall survival rate as obtained by the number of patients alive divided by the total patients at 60 months after surgery for ESCC and LACC was 72 of 76 (95%) and 37 of 45 (82%), respectively (hazard ratio, 5.4; 95% CI, 1.7-16.8; *P* = .001). The stage-specific 5-year overall survival for stage IB1, IB2, IIA, and IIB was 96% (95% CI, 88%-99%), 92% (95% CI, 57%-99%), 78% (95% CI, 37%-94%), and 78% (95% CI, 57%-89%), respectively. There was 1 patient with stage IB2 disease who died of intercurrent disease (primary lung cancer) at 105 months after surgery, and the 5-year disease-specific survival was the same as the 5-year overall survival. Univariable Cox regression analysis showed several risk factors associated with the death of patients. We had 16 death events, and we obtained a 2-variable regression model that included lymph node metastasis and histology (Table 4).

**Table 4.** Univariable and 2-Variable Cox Regression Analyses for the Risk of Decease After Nerve-Sparing Radical Hysterectomy^a^

Risk factor	Total No.	Death (%)	Cox regression analysis for risk factors of decease			
			Univariable, HR (95% CI)	*P* value	Two-variable model, HR (95% CI)	*P* value
2008 FIGO stage						
Early	76	4 (5)	1 [Reference]	NA	NA	NA
Locally advanced	45	12 (27)	5.4 (1.7-16.8)	.003	NA	NA
Histology						
Squamous	79	5 (6)	1 [Reference]	NA	1 [Reference]	NA
Adeno(squamous)carcinoma	42	11 (26)	4.7 (1.6-13.5)	.004	5.5 (1.9-16.1)	.003
pT classification						
pT1b	91	5 (5)	1 [Reference]	NA	NA	NA
pT2	30	11 (37)	8.3 (2.9-24.0)	<.001	NA	NA
Lymph node metastasis						
Negative	88	4 (5)	1 [Reference]	NA	1 [Reference]	NA
Positive	33	12 (36)	9.3 (3.0-28.8)	<.001	10.5 (3.4-32.8)	<.001
Lymphovascular space invasion						
Negative	67	5 (7)	1 [Reference]	NA	NA	NA
Positive	54	11 (20)	2.9 (1.0-8.4)	.05	NA	NA

Abbreviations: FIGO, International Federation of Gynecology and Obstetrics; HR, hazard ratio; NA, not applicable.

^a^ Univariable Cox regression analysis for overall survival was performed for each variable, and then a 2-variable regression model based on the number of events we observed was obtained.

## Discussion

We observed that Okabayashi-Kobayashi radical hysterectomy with adjuvant chemotherapy resulted in very good survival outcomes for ESCC (stage IB, ≤4 cm; stage IIA, ≤4 cm) and even in LACC (stage IB, >4 cm; stage IIA, >4 cm; stage IIB). Although the number of patients was limited, our study suggested favorable survival outcomes for stage IB2 disease treated with radical hysterectomy compared with published literature. Our finding supports the statement in the National Comprehensive Cancer Network Guidelines, in which type C1 (Querleu-Morrow) nerve-sparing radical hysterectomy is indicated for the new 2018 FIGO stages IB1 (<2 cm) to IB2 (2-4 cm) and selected stages IB3 (>4 cm) and IIA1. The 2018 FIGO stage IB3 (>4 cm) corresponds with the 2008 FIGO stage IB2 (>4 cm) without lymph node metastasis.^35^ Previous reports showed that the 5-year overall survival for stage IB2 disease treated with radical hysterectomy was 72% to 72.8%.^36,37^ Cost-effectiveness analysis for treatment of stage IB2 cervical cancer showed that upfront radical hysterectomy was the most cost-effective strategy compared with primary chemoradiotherapy or neoadjuvant chemotherapy followed by radical hysterectomy and adjuvant chemoradiotherapy.^38^ Our study suggested that Okabayashi-Kobayashi radical hysterectomy is applicable for this group of patients. A recent randomized clinical trial (EORTC 55994) for stage IB2-IIB cervical cancer showed equivalent overall survival for neoadjuvant chemotherapy followed by radical hysterectomy (72%; 95% CI, 66%-77%) and concurrent chemoradiotherapy (76%; 95% CI, 70%-80%) (*P* = .25). Additionally, a subgroup of patients with stage IB2 treated with neoadjuvant chemotherapy followed by radical hysterectomy showed a trend for better results compared with concurrent chemoradiotherapy.^39^

Our results showed that pathological vaginal and parametrial invasion and lymph node metastasis were closely associated with local recurrence after nerve-sparing radical hysterectomy. In a 1978 article,^40^ stage IIA disease had microscopic parametrial involvement at a risk higher than or equivalent to stage IIB disease. Perineural invasion is associated with deep cervical stromal, vaginal, and parametrial invasion.^41,42^ Magnetic resonance imaging findings of disruption of the cervical stromal ring are associated with microscopic parametrial invasion,^43^ and this finding might indicate an increased risk of nervous infiltration. We observed 4 cases of local recurrence in the paracolpium area on the same side as the nerve-sparing procedure, which suggested a possible relationship between the nerve-sparing method and local recurrence in some cases.

The feasibility of upfront surgical therapy for stage IIB disease using Okabayashi-Kobayashi radical hysterectomy^44^ or with extended mesometrial resection has been suggested.^45^ Our results of LACC can be discussed in relation to the 5-year overall survival from the Surveillance, Epidemiology, and End Results database^46^ and the 2006 FIGO Annual Report.^47^ The 5-year overall survival from the Surveillance, Epidemiology, and End Results database for stages IIA and IIB was 62% and 64%, respectively, and that from the 2006 FIGO report was 73% and 66%, respectively. The 5-year overall survival in our study for these stages was 78% and 78%, respectively. Our data appear to be consistent with the results from the Surveillance, Epidemiology, and End Results database and the FIGO Annual Report. Furthermore, the annual report of the committee on gynecologic oncology of Japan Society of Obstetrics and Gynecology on registered patients who were treated with surgery or radiotherapy in 2010 showed that the 5-year overall survival for stages IIA and IIB was 81% and 72%, respectively.^24^ The 5-year overall survival in our cohort corresponds with that in the Japan Society of Obstetrics and Gynecology report. We also compared our data with a nationwide multicenter study conducted by the Japan Gynecologic Oncology Group to evaluate the effect of surgical volume on survival after radical hysterectomy.^48^ Five-year DFS for stages IB1 to IIB disease combined in the Japan Gynecologic Oncology Group report was 77%, 80%, and 85% for the low-, mid-, and high-volume groups, respectively. Five-year DFS for stages IB1 to IIB combined in our cohort was 85%, which is consistent with that for the high-volume center in the Japan Gynecologic Oncology Group study.

The Okabayashi-Kobayashi method uses extensive excision of the vagina and paracolpium compared with the Meigs operation, which corresponds to the Piver class III operation. Meigs operation excises only the medial part of the ventral parametrium (anterior layer of the vesicouterine ligament).^49,50^ Complete separation of the bladder and ureter from the vagina and paracolpium seems difficult without excision of the vesicovaginal ligament (posterior layer of the vesicouterine ligament). The Piver class III operation and its equivalent methods would not likely deal with removing a sufficient extent of the vagina and paracolpium. Piver described that his class IV operation excises entire tissue surrounding the terminal ureter for extended excision of the vagina and the paracolpium, which corresponds to the Okabayashi-Kobayashi operation. The length of vaginal cuff removal less than 2 cm was suggested to be associated with increased local recurrence and decreased survival in stage IB-IIA cervical cancer.^51^ The surgical radicality for cervical cancer should include not only the extent of parametrial tissue, but also the extent of vaginal/paracolpium resection. We consider that more than 2 cm of vaginal cuff removal is critical for local control of LACC. Okabayashi-Kobayashi radical hysterectomy appears to be the choice of surgery for selected patients with LACC.

### Limitations

This retrospective study was done in a single tertiary care center, and it included a relatively small number of patients, which creates a limitation in terms of generalizability of the study. However, surgery was performed with well-standardized, systematic, nerve-sparing Okabayashi-Kobayashi radical hysterectomy. Additionally, an intensive effort was made to ensure that the quality of follow-up was as high as possible, with no cases lost to follow-up at less than 60 months.

## Conclusions

The nerve-sparing Okabayashi-Kobayashi radical hysterectomy for LACC may provide survival not inferior to radical hysterectomy or radiotherapy described in the published literature. The applicability of radical hysterectomy with adjuvant chemotherapy for LACC needs to be validated by prospective comparative trials.
